# Detection and genome characterization of Middelburg virus strains isolated from CSF and whole blood samples of humans with neurological manifestations in South Africa

**DOI:** 10.1371/journal.pntd.0010020

**Published:** 2022-01-03

**Authors:** Isabel Fourie, June Williams, Arshad Ismail, Petrus Jansen van Vuren, Anton Stoltz, Marietjie Venter

**Affiliations:** 1 Zoonotic Arbo-and Respiratory Virus (ZARV) program, Centre for Viral Zoonoses (CVZ), University of Pretoria, Pretoria, South Africa; 2 Department of Paraclinical Sciences, Faculty of Veterinary Science, University of Pretoria, Onderstepoort, South Africa; 3 Sequencing Core Facility, National Institute of Communicable Diseases (NICD), Division of National Health Laboratory Service (NHLS), Sandringham, South Africa; 4 Australian Centre for Disease Preparedness, CSIRO-Health and Biosecurity, Geelong, Australia; 5 Infectious diseases, Steve Biko Hospital, Pretoria, South Africa; University of Geneva Hospitals, SWITZERLAND

## Abstract

**Background:**

The Old world *Alphavirus*, Middelburg virus (MIDV), is not well known and although a few cases associated with animal illness have previously been described from Southern Africa, there has been no investigation into the association of the virus with human illness. The current study aimed to investigate possible association of MIDV infection with febrile or neurological manifestations in hospitalized or symptomatic patients fromGauteng, South Africa.

**Methods:**

This study is a descriptive retrospective and prospective laboratory based study. Archived cerebrospinal fluid (CSF) samples submitted to the National Health Laboratory Service (NHLS), Tshwane Academic division for viral investigation from public sector hospitals in Gauteng as well as EDTA (ethylenediaminetetraacetic acid) whole blood samples from *ad hoc* cases of veterinary students, presenting with neurological and febrile illness, were selected and screened for the presence of alphaviruses using real-time reverse transcription(rtRT) PCR.Virus isolations from rtRT-PCR positive samples were conducted in Vero cell culture and used to obtain full genome sequences. Basic descriptive statistical analysis was conducted using EpiInfo.

**Results:**

MIDV was detected by rtRT-PCR in 3/187 retrospective CSF specimens obtained from the NHLS from hospitalised patients in the Tshwane region of Gauteng and 1/2 EDTA samples submitted in the same year (2017) from *ad hoc* query arbovirus cases from veterinary students from the Faculty of Veterinary Science University of Pretoria.Full genome sequences were obtained for virus isolates from two cases; one from an EDTA whole blood sample (*ad hoc* case) and another from a CSF sample (NHLS sample).Two of the four Middelburg virus positive cases,for which clinical information was available, had other comorbidities or infections at the time of infection.

**Conclusion:**

Detection of MIDV in CSF of patients with neurological manifestations suggests that the virus should be investigated as a human pathogen with the potential of causing or contributing to neurological signs in children and adults.

## Author summary

Old World alphaviruses are mainly associated with polyarthritis in humans. Global emergence of chikungunya virus raises concerns regarding disease potential of neglected African arboviruses.New World alphaviruses are associated with severe neurological disease in humans and horses in the Americas with a high mortality rate. Middelburg virus (MIDV) is an Old World alphavirus previously associated with neurological disease and fatalities in horses and wildlife in southern Africa but not yet linked to disease in humans. This study investigated MIDV in humans presenting with acute neurological symptoms in South Africa. MIDV infection was confirmed in 4/189 (2%) such cases using a generic *Alphavirus* real-time RT-PCR and phylogenetic analysis. MIDV full genomes were obtained from a cerebrospinal fluid sample of a 2-year-old child and a whole blood sample of a veterinary student, both presenting with neurological signs. This study suggests MIDV may be associated with unsolved neurological disease in humans in Africa.

## Introduction

Middelburg virus (MIDV) is a member of the *Alphavirus* genus, family *Togaviridae* [1]. The alphaviruses currently include more than 30 species divided into 11 antigenic complexes based on serological cross-reactivity and genetic data [1]. The antigenic complexes mostly correlate with Old and New World locations and clinical signs although members within the same antigenic complex can differ in geographic location.

The New World alphaviruses are mostly restricted to the Americas and include western-(WEEV), eastern (EEEV)- and Venezuelan equine encephalitis (VEEV) viruses that are associated with more severe disease and encephalitis in humans and animals, especially in equids [2–7]. Old World alphaviruses such as o’nyongnyong (ONNV)-, CHIKV-,Ross River (RRV) and Sindbis virus (SINV) are traditionally restricted to Africa, Eurasia and Australia [8,9] and are mainly associated with rash and arthritis with symptoms often persisting for years after infection [8,10,11]. Although neurological symptoms associated with CHIKV were relatively uncommon, reports of neurological manifestations and fatalities in children and adults associated with CHIKV infection from India, La Reunion Island and the Caribbean have increased in the past decade [12–14].

Middelburg virus, like other alphaviruses, is an enveloped virus with a single stranded positive sense RNA genome of ~11.67-kb, a poly (A) tail, a 5’ terminal cap and a 3’UTR. Open reading frame (ORF) one consists of non-structural proteins 1–4 (nsP) and the 26S junction region; ORF two consists of the structural proteins (sP) C, E3, E2,6K and E1 [15]. MIDV was first isolated from *Ae*. *caballus* and *Ae*. *(Banksinella) sp*. mosquitoes in the Eastern Cape Province of South Africa in 1957 [16]. Experimental inoculation of the mosquito MIDV isolate resulted in rapid febrile response in lambs and fatalities in mice [16]. Neutralizing antibodies against MIDV were also detected in human serum from individuals involved in mosquito collections during the outbreak, but were not reported to be associated with disease [16].Since its discovery MIDV has been isolated from different *Aedes (Ae*.*) sp* mosquitoes across Africa as well as from a tick (*Amblyomma variegatum*) [17–19] but only *Ae*. *caballus* has been shown to successfully transmit MIDV horizontally to lambs [16].

The first indication that MIDV could cause severe disease in animals was following the isolation of MIDV from the spleen of a horse in Zimbabwe that died in 1993 [20] after presenting with clinical signs resembling African Horse sickness virus infection. Signs included a rise in body temperature, tachycardia, pulmonary involvement, and oedema particularly in the head and neck [20]. Different studies from South Africa between 2008–2013 have further identified MIDV and SINV infections associated with febrile and neurological disease in horses [21] and wildlife[22], including fatalities, with MIDV being detected in brain specimens of a number of the horses.

The data from Southern Africa has shown the potential of MIDV to cause severe illness not only in horses but also in several wildlife and livestock species suggesting a wide host range [21–23]. Historic reports of seropositivity in humans suggest zoonotic potential for MIDV [16]. There is currently no information on the disease potential of MIDV in humans. This study aimed to investigate Middelburg virus and other alphaviruses’ possible association with acute neurological cases in humans in the Gauteng province (South Africa) where numerous horse cases were previously detected.

## Materials and methods

### Ethical statement

Ethical approval was obtained from the University of Pretoria’s Research Ethics Committee, project number 441–17. Informed written consent was obtained directly from the veterinary student while written permission was obtained through the *National Health Laboratory Service* (NHLS) and from individual hospitals where specimens originated to retrospectively collect CSF specimens and access patient data respectively.

### Sample selection

CSF: A random selection of cerebrospinal fluid (CSF) samples submitted to the *National Health Laboratory Service*(NHLS); Tshwane Academic division were kindly archived by NHLS staff to allow for arboviral investigation. The samples were collected for the NHLS for viral investigation as requested by the treating physician. Samples were collected from patients presenting with acute febrile/neurological manifestations from public sector hospitals in Gauteng by qualified medical personnel at these respective hospitals. 187 CSF samples collected between February to May 2017 were included for arbovirus analysis. Samples from males and females off all age groups were included in analysis regardless of other diagnosis.

EDTA: Two EDTA (ethylenediaminetetraacetic acid) whole blood samples from *ad hoc* cases of veterinary students, presenting with neurological and febrile illness, were included. Specimens were again taken during the typical arbovirus season, February- May, of 2017.

### Data collection and analysis

All data were entered into a database, all names and identifiers were excluded for further analysis and publications.Tests requested by the treating clinician and results found through diagnostic virology testing by the NHLS lab were also recorded in the database.Samples were grouped into four categories based on age: age groups 1 (0–5 years), 2 (6–18 years), 3 (19–59 years) 4 (60 years and above).

### Viral detection and sanger sequencing

RNA was extracted from CSF and EDTA whole blood using the QIAmp viral RNA Kit, (Qiagen, Valencia, CA, USA) as per manufacturer’s instructions. Extracted RNA was subjected to screening using a published nested real-time alphavirus PCR with probes specific for Middelburg and Sindbis virus [21,24]. Primers/probes used in the current study are summarized in S1 Table and method described in S1 Text. PCR products were also visualised on an agarose gel to detect all alphaviruses by the generic alphavirus primers. The limit of detection was 10 RNA copies per ml for both MIDV and SINV in the second (nested) reaction which was determined by testing serial dilutions containing 7.36 x 10^7^ to 7.36 x 10^−1^ and 6.62 x 10^7^ to 6.62 x 10^−1^ copies per ml of transcribed RNA for MIDV and SINV respectively. The positive cut off value was determined as Ct value ≤ 38.

In order to improve phylogenetic analysis a larger fragment of 347 base pairs (bp) of the nsP4 gene was amplified using MIDV specific primers [22] for all MIDV real time PCR positive samples. A 550 bp fragment of the MIDV E1 gene was also amplified using a combination of newly designed and previously published[21] primers to investigate recombination events (S1 Table).

For differential diagnosis all MIDV positive specimens were also screened for flaviviruses and Shuni virus [25,26] and the veterinary students were also tested on a meningochip macroarray for common and zoonotic causes of febrile and neurological disease [27].

### Virus isolation and Sequence-Independent Single-Primer Amplification (SISPA) with Rapid Amplification of cDNA Ends (RACE)

Virus isolation from PCR positive CSF or EDTA blood samples was attempted using Vero E6 cells in the BSL-3 facility at the University of Pretoria’s Centre for Viral Zoonosis (CVZ) (S1 Text). Extracted concentrated RNA was converted to cDNA and amplified using the modified SISPA (RACE) technique described previously to enhance coverage of terminal ends [28].

### Full genome sequencing

Synthesised cDNA was sent to NICD Sequencing Core Facility (National Institute for Communicable Diseases, Sandringham, South Africa) for sequencing. Paired-end libraries were prepared using the Nextera DNA Flex library preparation kit, followed by sequencing (2 x 300 bp) on a MiSeq instrument (Illumina, San Diego, CA, USA)

### Phylogenetic analysis

Sequences of other members of the *Alphavirus* genus were obtained from NCBI-Nucleotide database (GenBank) and accession numbers are indicated in S2 Table. Sanger sequenced nsP4 and E1 fragments and full genome sequences were analysed using CLC main workbench v8 (https://www.qiagenbioinformatics.com/). For full genomes failed reads and low-quality scores were discarded and reads mapped against MIDV SAE25/11 (GenBank accession number KF680222.1) as reference sequence. The reference sequence was obtained from a previous MIDV positive South African horse that presented with febrile illness [21]. For improved phylogenetic analysis, the two ORFs were obtained from full genome sequences for currently described MIDV strains and concatenated. Alphavirussequences from the different antigenic complexes were downloaded from Genbank (S2 Table) and ORFs obtained in the same manner. Multiple sequence alignments were performed using the online MAFFT v7 alignment tool available on https://mafft.cbrc.jp/alignment/software/[29]. The C terminal of the nsP3 and the N terminal of the capsid sequences were removed to allow for better phylogenetic analysis as described by previous authors since these regions contain extensive divergence hindering reliable alignments [30,31]. Optimal evolutionary trees were estimated using jModelTest2 v2.1.6 [32] and maximum-likelihood (ML) analysis conducted using BEAST v1.10.4 [33] accessed through the CIPRES Science Gateway [34] utilizing a GTR+G+I model and uncorrelated relaxed clock. Phylogenetic analyses were conducted for the nsP4 and the E1 fragment amplicons of the four currently described strains. The two full genome sequences were subjected to three separate phylogenetic analyses using the nsPs, the sPs and the concatenated ORF1 and ORF2 (thus full coding region). Pairwise distance (p-distance) analysis was calculated in MEGA 7 (Molecular Evolutionary Genetics Analysis software) [35] and used to determine percentage identities between different MIDV strains. Sequences were submitted to GenBank and raw sequence data for full genomes were submitted to the NCBI Sequence Read Archive (Accession numbers are as follows ZRU099/17:MN967314 and SAMN22891582; ZRUH399/17:MN967313 and SAMN22891583; ZRUH177/17 nsP4: MT264776 and E1: MT264774; ZRUH248/17: nsP4: MT264777 and E1 MT264775.

### Recombination analysis

The recombination analysis program RDP4 [36]; which alongside RDP[37] also uses other recombination analysis programs including GENECOV [38], Chimaera [39], Sciscan [40], Bootscan [41], MaxChi [42] and 3Seq [43]; was used to detect recombination events in the structural region of alphavirus sequences.

## Results

### Virus detection

Real-time alphavirus genus RT-PCR with Middelburg and Sindbis virus specific probes identified Middelburg virus in 3/187 CSF specimens collected through the NHLS and one of two EDTA samples submitted in the same year (2017) from *ad hoc* query arbovirus cases from veterinary students. Most of the samples in the sample group were from children 0–5 years (83/189, 44%) and adults 19–59 years (72/189, 38%), followed by age group 6–18 years (26/189, 14%) and age group >60 years (8/189, 4%). Three of the four MIDV positive cases were detected in adults (19–59 years) and one case in a two-year-old child. MIDV cases were detected equally (50%) in males and females.

No other alphaviruspositives were detected during screening and all samples were negative for Shuni virus and flaviviruses. Ct values ranging between 21 and 38 were obtained for MIDV positives with the nested real-time(rt) RT-PCR [21]. Virus isolation was attempted for all four rtRT-PCR MIDV positive samples in the BSL-3 laboratory and was successful for one EDTA sample (ZRU099/17, *ad hoc* student) and one CSF sample (ZRUH399/17, NHLS). Success of virus isolations was determined by detection of MIDV via real-time PCR on the first round reaction described above.

### Description of positive cases

The patient demographics and clinical signs are summarised in Table 1. Neurological signs were present in all MIDV positive patients for whom clinical details were available but varied between patients.

**Table 1:** Patient information for Middelburg virus positive human cases.

Sample ID	Sample collection and type	Age	Sex	Symptoms	Real-time PCR Ct value	Other diagnosis	HIV status	Other tests (result)	Location
ZRU 099/17	*Ad hoc* veterinary student EDTA	24	F	Cough, fever, neck stiffness, myalgia, nausea, severe headaches	37.51	None (history of brain tumour, in remission, no treatment at suspected time of infection)	(-)	Meningochip (31 pathogens Chipronmacroarray)[27] (all (-)) Other alphaviruses (-) Shuni virus (-) Flaviviruses (-)	Vereeniging (weekends) Onderstepoort veterinary Faculty, Pretoria (weekdays)
ZRUH 177/17	Retrospective NHLS sample CSF	49	M	Acute blindness, general weakness, body pains	34.65	VZV, syphilis, Devic’s disease	(+)	VZV (+) syphilis (+) JC-virus (-) Other alphaviruses (-) Shuni virus (-) Flaviviruses (-)	Small holding Zwavelpoort, Pretoria East, Gauteng
ZRUH 248/17	Retrospective NHLS sample CSF	30	F	Unknown	21.22	Unknown	Unknown	HSV 1& 2 (-) Other alphaviruses (-) Shuni virus (-) Flaviviruses (-)	Unknown
ZRUH 399/17	Retrospective NHLS sample CSF	2	M	Tonic-clonic seizures, diarrhoea, vomiting	34.57	Acute gastric enteritis, dysentery	(-)	Enterovirus (-) Shigella dysentery (+) Other alphaviruses (-) Shuni virus (-) Flaviviruses (-)	Centurion, Gauteng

(-): negative result, (+): positive result: CSF: cerebrospinal fluid; EDTA: ethylene diamine tetra acetic acid; HSV: Herpes simplex virus; JC: John Cunningham virus; NHLS: National Health Laboratory Service; VZV: Varicella zoster virus

### NHLS CSF specimens

Patient information was only available for two of the three MIDV positive cases collected through the NHLS. The first was a 49-year-old Human Immunodeficiencyvirus (HIV) positive male admitted to hospital with general weakness and body pains, acute blindness, and HIV- associated peripheral neuropathy with a CD4 count of 26. The patient had been on HAART(Highly Active Antiretroviral Therapy) for approximately one year and was diagnosed with Meningitis at the time of admission. The CSF sample also tested positive for varicella-zoster virus (alphaherpesvirus) and a serum sample from the patient tested positive for syphilis (Treponema pallidum subspeciespallidum) according to the laboratory records. Treatment included HAART, Rocephin as short course antibiotic followed by a longer course of Acyclovir(antiviral) and Penicillin G treatment. The patient was released to a rehabilitation centre following stabilization and vision improvement. He resides on a smallholding in Pretoria, which is in a rural area outside the city.

The second sample was from an HIV negative 2-year-old boy who was admitted to hospital for tonic-clonic seizures, fever, diarrhoea, and vomiting. The boy had no family or personal history of seizures or epilepsy. Meningitis was investigated by a CT scan and enterovirus, (Picornaviridae family) PCR which was negative. A clinical diagnosis of acute gastroenteritis was confirmed by positive blood culture of Shigella dysenteriae, which correlates to the observed symptoms [44]. The CSF sample was not tested for the presence of Shigella. The boy recovered and was discharged 3 days later. He was residing in Centurion, (situated just south of Pretoria) at the time of hospitalization. EDTA sample from ad hoc veterinary student:

A 24-year-old HIV negative female veterinary student with the following history: diagnosed with an ependymoma at the posterior fossa in 2013, which was removed by craniotomy. In 2015 she relapsed and received intense radiation until January 2016 and in 2017 she was hospitalised for treatment of radiation side effects. During 2017 she presented to a visiting campus doctor with a cough, fever, neck stiffness, myalgia, nausea, and severe headaches. She was clinically diagnosed and treated for tick bite fever although this was not confirmed with laboratory tests and no skin lesions were identified. An EDTA sample taken two weeks after onset of initial symptoms tested positive for MIDV by real-time RT-PCR and negative for 31 other pathogens associated with febrile and neurological signs (S3 Table) using a macroarray[27]. A CSF sample collected 29 days after onset of symptoms and 16 days after the MIDV PCR positive blood sample was taken but tested negative for MIDV at this point. Three months after the initial onset of symptoms she again presented with the same symptoms, with additional light sensitivity and a pruritic rash on the legs, arms, stomach and back at which time the blood sample was PCR negative for MIDV. At the suspected time of infection, she resided at a residence on the Onderstepoort veterinary campus of the University of Pretoria during the week and stayed on a small holding in Vereeniging, south of Johannesburg, Gauteng province over weekends. She had minimal direct contact with livestock, domestic and wild animals although these were present on the same campus. She reported being bitten by mosquitoes while residing at the residence. One of the horses kept in the veterinary campus paddocks close to her residence presented with febrile, and neurological signs and tested positive for MIDV during this period. Following the MIDV infection she made a full recovery but has had other undiagnosed health related issues since.

### Phylogenetic analysis

**Fig 1:**
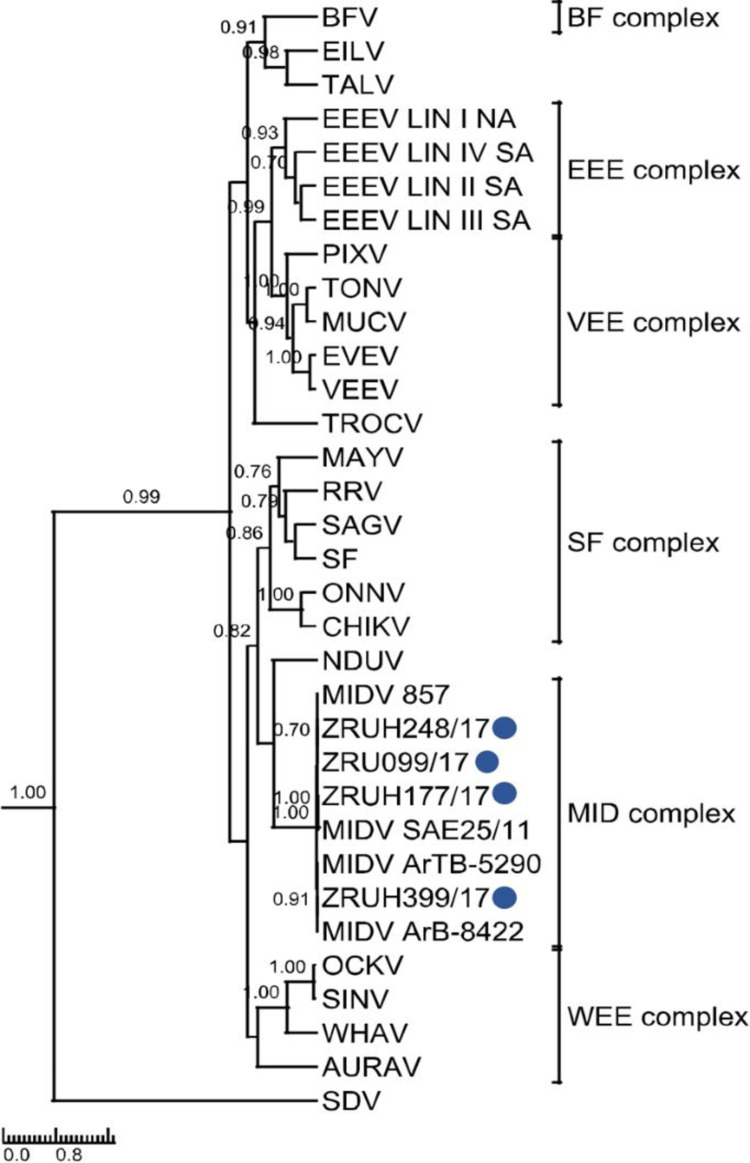
Rooted phylogenetic tree of 347base pair nsP4 fragment produced from Bayesian analysis using BEAST. Analysisof 33 taxa, model GTR+G+I of alphaviruses is shown. Posterior probabilities >0.7 are shown on major branches. Middelburg virus human positives described in current study indicated with ZRU numbers and blue circles. Alphavirus complexes indicated by brackets; tree is drawn to scale representing the number of nucleotide substitutions per site.

**Fig 2:**
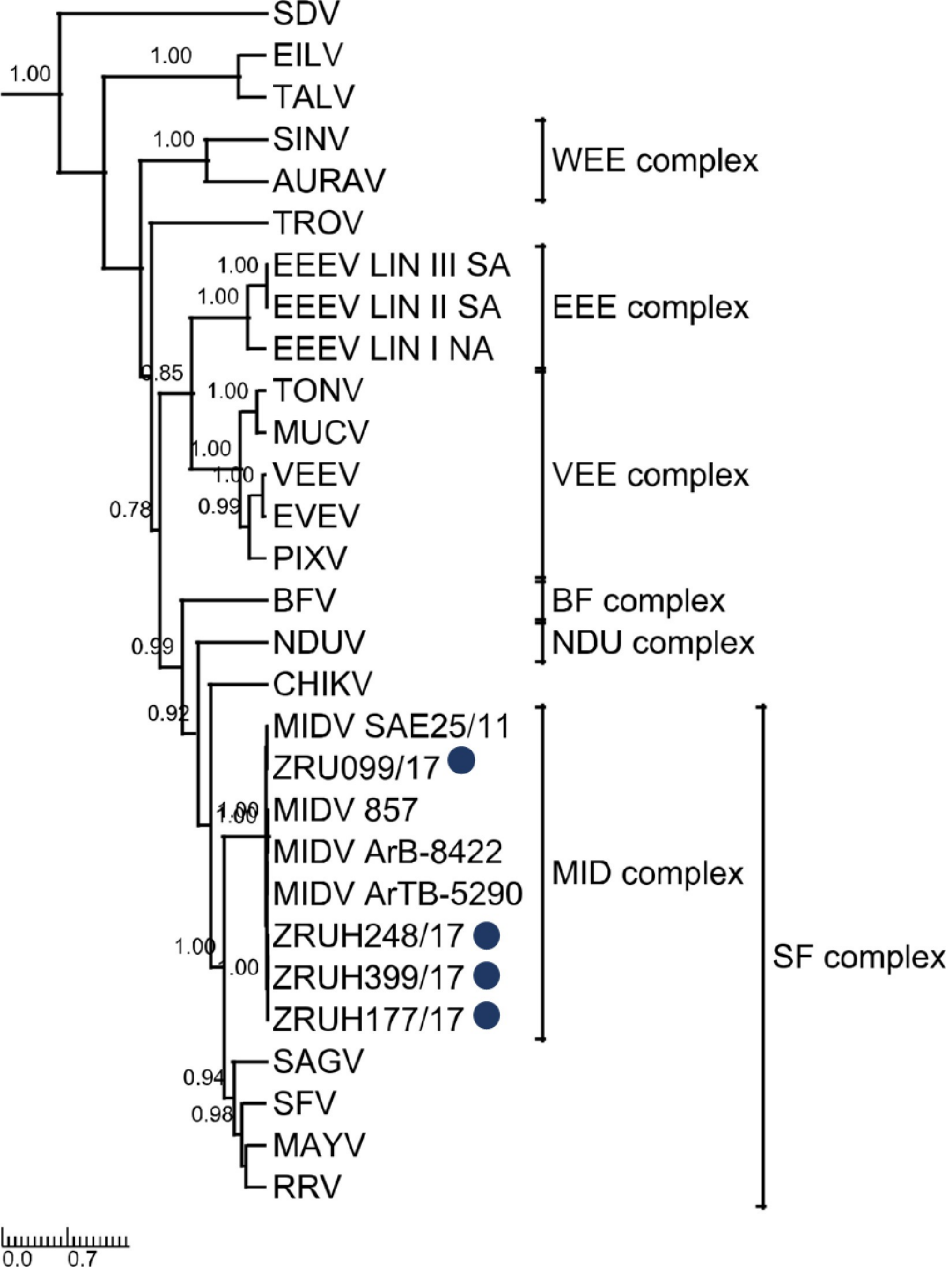
Rooted phylogenetic tree of 550 base pair E1 fragment produced from Bayesian analysis using BEAST. Analysis of 29 taxa, model GTR+G+Iof alphaviruses is shown. Posterior probabilities >0.7 are shown on major branches. Middelburg virus human positives described in the current study are indicated with ZRU numbers and blue circles. Alphavirus complexes are indicated by brackets. The tree is drawn to scale representing the number of nucleotide substitutions per site. Full genomes were obtained for ZRU099/17 (EDTA whole blood sample, *ad hoc*veterinary student) and ZRUH399/17 (CSF sample obtained through NHLS from 2 year old child) following virus culturing on Vero cells for both samples and an unbiased next-generation sequencing approach using SISPA-RACE amplification. Assembled contigs for ZRU099/17 and ZRUH399/17 were mapped to MIDV SAE25/11 resulting in full genomes of 11 673 nucleotides (nt) in length, excluding the poly (A) tail. P-distance analysis for full genomes showed the greatest percent identity with the strain isolated from the spleen of the horse from Zimbabwe (MIDV 857), that presented with AHSV-like signs and the South African strain (MIDV SAE25/11) with even greater percentage identities between the two human strains (S4 Table). The concatenated ORF1 and ORF2 p-distances resulted in 99.48% and 99.51% amino acid (aa) identities for ZRU099/17 and ZRUH399/17 compared to MIDV SAE25/11, respectively. Phylogenetic analysis with strong posterior probability support was obtained as follows: the concatenated (ORF1 and ORF 2) and nsP region placed MIDV in its own complex as seen for analysis using nsP4, while analysis using the sP region placed MIDV within the SF complex as seen with analyses involving the E1 fragment (which forms part of the structural proteins) (Figs 3, 4 and 5). Sequences for both ZRU099/17 and ZRUH399/17 contained the 3’UTR repeats that have been identified in MIDV strains isolated from horses but absent from arthropods (S1 Fig) [19–21]. The function of these repeats is still unknown but it has been hypothesized to play a role in virus adaptation to vectors [45]. S5 and S6 Tables show a summary of amino acid and nucleotide changes between currently described and previously published MIDV strains in the ORF’s and UTR’s, respectively. Only positions where changes between MIDV SAE25/11 and other MIDV strains occurred are indicated since MIDV SAE25/11 is a previously identified South African strain. Changes between ZRU099/17 and ZRUH399/17 compared to MIDV SAE25/11 are highlighted in grey.

Phylogenetic analysis of the 347 bp nsP4 fragment (Fig 1) as well as the 550 bp E1 fragment (Fig 2) clustered the human MIDV strains with previously detected MIDV strains from horses and arthropods. Analysis of the nsP4 region placed MIDV in its own complex with other MIDV strains while that of E1 placed it within the Semliki Forest (SF) complex. P-distance analyses ranged from 97–99% for all four samples in both the nsP4 and E1 amplicon regions as compared to previously published MIDV strains (S4 Table).

**Fig 3:**
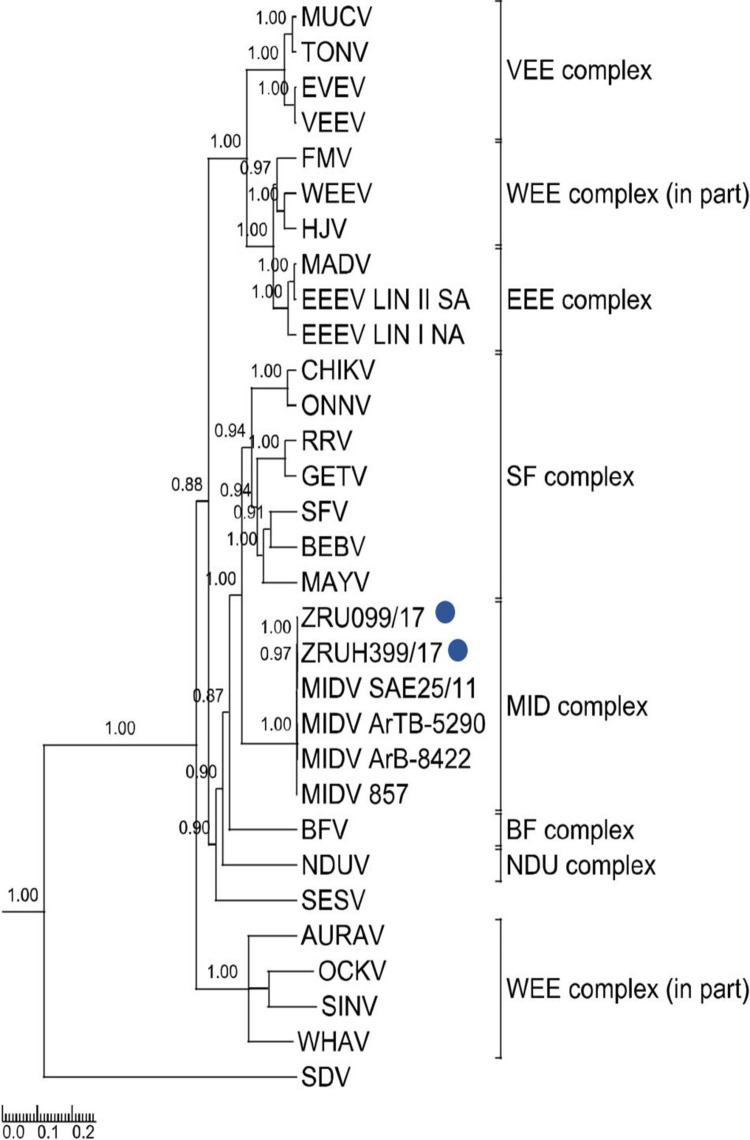
Rooted phylogenetic tree produced from Bayesian analysis using BEASTon 7236 base pair non-structural proteins. Analysis of 31 taxa, model GTR+G+I of alphaviruses is shown. Posterior probabilities >0.7 are shown on major branches. Sequences of human Middelburg virus positives described in the current study are indicated with ZRU numbers and blue circles. Alphavirus complexes are indicated by brackets. The tree is drawn to scale representing the number of nucleotide substitutions per site. (31 taxa, model GTR+G+I) of alphaviruses is shown. Posterior probabilities >0.7 are shown on major branches. Sequences of human Middelburg virus positives described in the current study are indicated with ZRU numbers and blue circles. Alphavirus complexes are indicated by brackets. The tree is drawn to scale representing the number of nucleotide substitutions per site.

**Fig 4:**
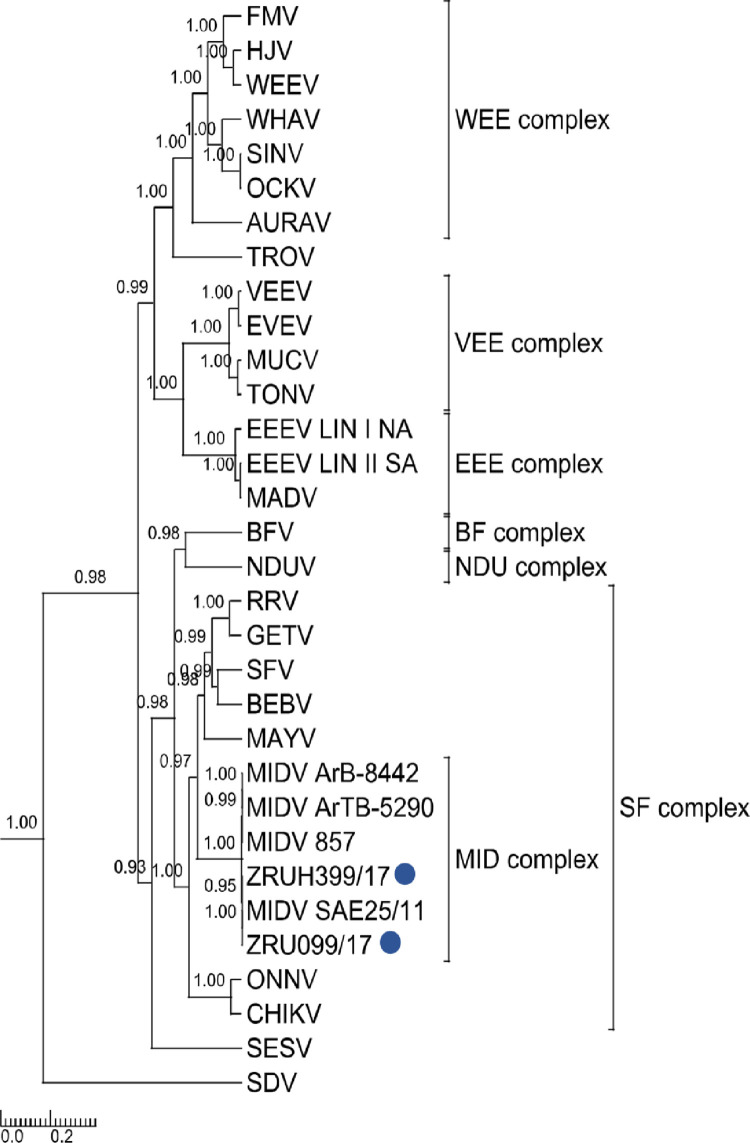
Rooted phylogenetic tree produced from Bayesian analysis using BEAST on 3777 base pairstructural proteins. Analysis of32 taxa, model GTR+G+I of alphaviruses is shown. Posterior probabilities >0.7 are shown on major branches. Sequences of human Middelburg virus positives described in the current study are indicated with ZRU numbers and blue circles. Alphavirus complexes are indicated by brackets. The tree is drawn to scale representing the number of nucleotide substitutions per site.

**Fig 5:**
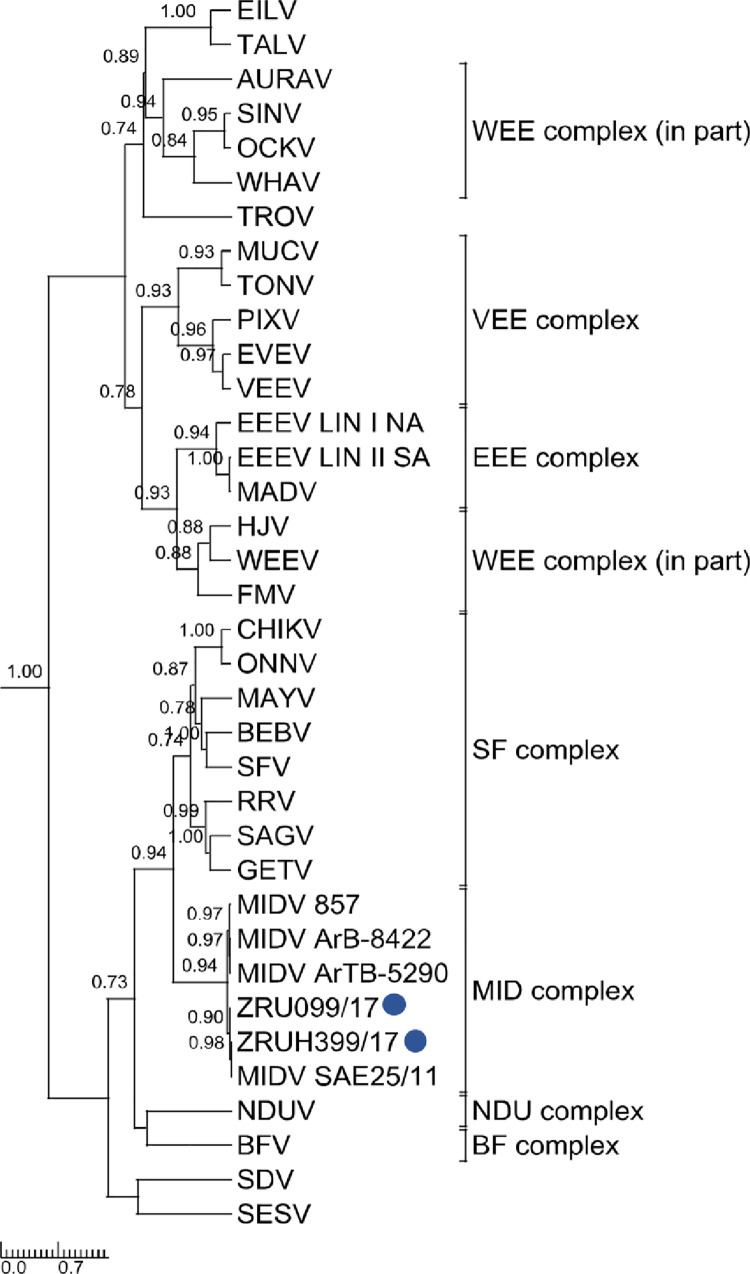
Rooted phylogenetic tree produced from Bayesian analysis using BEASTon non-structural and structural protein sequences. Analysis of 36 taxa, model GTR+G+I of alphaviruses is shown. Probabilities >0.7 are shown on major branches. Sequences of human Middelburg virus positives described in the current study are indicated with ZRU numbers and blue circles. Alphavirus complexes are indicated by brackets. The tree is drawn to scale representing the number of nucleotide substitutions per site.

### Recombination event analysis

**Fig 6:**
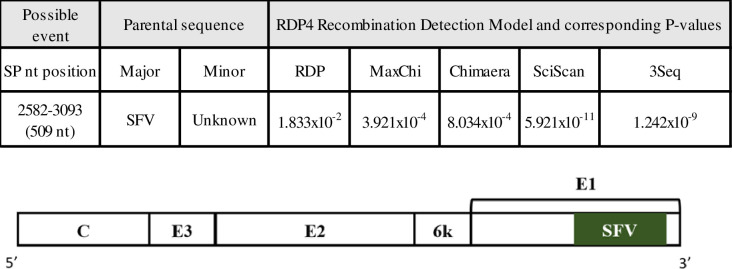
Schematic representation of possible recombination events within structural regions of ZRU099/17 and ZRUH399/17. Possible recombination events within the structural region) for ZRU099/17 and ZRUH399/17 are shown.Only detection models with significant statistical support (p<0.05) are shown. Different genes encoding for the structural protein are indicated with the recombination event indicated in the green block. Nucleotide position corresponds to that of ZRU099/17. SFV Semliki Forest Virus (Genbank accession number CAA27742); nt = nucleotide.

Using the RDP4 program potential recombination events were detected in both currently described full genome MIDV strains isolated from humans involving an event in the structural protein region (p<0.05) at nucleotide position 2582–3093 (509 nt) of the structural protein (nucleotide position according to ZRU099/17) (Fig 6 and S2). Semliki Forest virus was identified as a potential major parent, while the minor parent remained unknown. The recombination event was detected by fiveout of the nine (RDP, MaxChi, Chimaera, SciScan and 3Seq) detection models used during the current analysis within the RDP4 program.

## Discussion

The traditional view of Old World alphaviruses being associated with less severe symptoms than the New World alphaviruses has been challenged by cases of CHIKV infection resulting in severe neurological manifestations [46–51]. The Old World MIDV and SINV alphaviruses have also been shown to be associated with severe neurological manifestations and even fatalities in horses and other animals from southern Africa [21,23]. The investigation of MIDV as a cause for undetermined neurological disease in humans could provide valuable information of the clinical presentation of a relatively unknown Old World alphavirus.

Serological studies at the time of its discovery suggested MIDV may be able to infect humans [16]. The current study reports the RT-PCR detection of Middelburg virus from three human CSF and one whole blood samples where patients presented with neurological manifestations and full genome sequence analysis of respectively one virus cultured CSF and EDTA sample.

All MIDV positives for which clinical data was available also had other infections/conditions and although Middelburg virus was not the only pathogen identified in these cases, MIDV was detected in the CSF of three patients. This suggests MIDV was able to cross the blood-brain barrier which may contribute to the severity of observed neurological manifestationsin these patients. One of the MIDV positives detected in a CSF sample was a HIV positive male. Given the high burden of HIV in a South African setting [52] a large proportion of the population is immunocompromised and thus may be more vulnerable to severe disease following viral infections and potentially change the pathogenic potential for previously neglected endemic arboviruses.

Clinical signs described in the veterinary student specifically was typical of an arbovirus infection with arthralgia and neck stiffness although this information was not available for the other patients.This patient again presented with the same signs and symptoms with an additional rash three months after her MIDV positive result. Infection with Old World alphaviruses, SINV and CHIKV, have been reported to result in prolonged joint pains and arthritis [10,11,53] and, less frequently reported, reoccurring rash, fatigue, myalgia and neuritis [54].MIDV was also detected in horses with neurological signs within the Gauteng region where these human positives were detected duringthe same period (Reported elsewhere).

The use of PCR for diagnosis has led to the extension of the clinical phenotype of numerous viral infections, including herpes simplex type 2 (HSV-2) and varicella zoster virus (VZV) which are now known to be common causes of aseptic meningitis even without a rash[55–57]. Detection of viral infection using PCR has been particularly useful in settings where multiple viruses are endemic to the region, complicating diagnosis based on clinical manifestations, as is the case for ZIKV, DENV and CHIKV [58].

Phylogenetic analysis based on concatenated ORFs, nsPs and the partial nsP4 fragment placed MIDV in a separate complex with strong posterior probability support. However, the partial E1 fragment and sP’s analysis placed MIDV within the SF complex. Recombination analysis using the RDP4 program also revealed potential recombination events on the E1 region with SF as the potential “major parent” sequence donor. This correlates with what has previously been described for MIDV as it has been suggested that MIDV originated from a recombination event between members of the SF complex[20]. The full-length genome analysis is believed to provide the most accurate placement of MIDV as it is based on both ORFs and is more consistent with serological relationships [30].

Limitations of this study include but are not limited to the lack of a control group,limited clinical information for negative samples and one MIDV positive sample, absence of serological assays and possible cell culture sequence bias. Although a control group would have been valuable, we do not have an ethical means to obtain CSF from patients if not for diagnostic purposes.The samples in this study were retrospectively collected from a diagnostic lab thus no controls were available. The low detection rate in CSF suggest however that this is not a common occurrence.

Interpretation of serological assays, such as Neutralization assays, for diagnosis of alphaviruses can be complicated due to cross-reaction between different members of the alphaviruses, especially within the same antigenic complex [24]. These tests are also less sensitive and more time consuming than newer molecular biology methods, such as the polymerase chain reaction (PCR)[24,59].Addition of IgM serological assays may however help to determine the true burden of disease in cases that had passed the viraemic phase. Culture isolates were used for NGS to improve the chances of obtaining full genome sequences since MIDV could only be detected in these samples via a nested PCR. This is likely due to declined viral load at the time of sampling as these patients were most probably infected with MIDV prior to sampling for viral investigation as other causes of signs and symptoms were first investigated.Thus, cell culture bias cannot be excluded. However, the MIDV strains obtained in the current study from human samples are similar to those obtained in previous studies from horse and arthropod samples [19–21].

Future investigations will aim to identify cases in both mild (febrile) cases with sera samples and more severe cases with CSF as part of a prospective active surveillance cohort study where we are enrolling patients with fevers of unknown origin with or without neurological signs.

This study was not structured to provide definitive information on the prevalence and incidence of Middelburg virus in humans. Although the clinical relevance of detection of MIDV in CSF of patients with neurological manifestations does suggest that MIDV should be investigated as a human pathogen with the potential of causing neurological signs in children and adults, particularly immune suppressed individuals, which may exacerbate disease in the African setting. MIDV should also be investigated elsewhere in Africa and in cases of fever of unknown cause to define the disease spectrum.

## Supporting information

S1 Text Methods. (DOCX)

S1 Table Primers and probes used for detection of alphaviruses targeting a conserved region of the nsP4 gene in alphaviruses and the E1 region of Middelburg virus specifically. (DOCX)

S2 Table GenBank and NCBI Sequence read archive accession numbersfor sequence data for members of the *Alphavirus* genus used for phylogenetic analysis in this study. Sequences used in the current study are in bold font.
(DOCX)

S3 Table Pathogens detected on the Chipron microarray. (DOCX)

S4 Table Percentage identity of human Middelburg virus isolate full genomes and individual proteins to previously identified Middelburg virus strains. Nucleotide and amino acid identities are shown for complete (concatenated) genomes with amino acids in the lower left matrix and nucleotides in the upper right matrix. Only amino acid identities are shown for individual proteins.
(DOCX)

S5 Table Amino acid comparisons of the structural and non-structural proteins between different Middelburg virus strains. Only positions where changes occurred as compared to MIDV SAE25/11 are indicated. Numbering refers to sequence positions of isolate SAE25/11. Changes in ZRU099/17 and ZRUH399/17 as compared to MIDV SAE25/11 are highlighted in grey.
(DOCX)

S6 Table Nucleotide acid comparisons of the 5’ and 3’ non-coding regions between different Middelburg virus strains. Only positions where changes occurred as compared to MIDV SAE25/11 are indicated. Numbering refers to sequence positions of isolate SAE25/11. Changes in ZRU099/17 and ZRUH399/17 as compared to MIDV SAE25/11 are highlighted in grey. Gaps and inserts are indicated with “-” and “^” symbols, respectively.
(DOCX)

S1 Fig The human MIDV strains, ZRU099/17 and ZRUH399/17 contain repeat sequences in the 3’UTR as observed in MIDV strains from horses (MIDV 857 and MIDV SAE25/11) but missing from historical arthropod strains (MIDV ArB-8422 and MIDV ArTB-5290) as indicated by the outlined box. “_” indicates absent sequence. MIDV = Middelburg virus.
(DOCX)

S2 Fig Screenshots of the identified possible recombination events associated with currently described MIDV full genomes (ZRU099/17 and ZRUH399/17)) within the structural region. A) UPMGA phylogenetic tree indicating the breakpoint region with probabilities shown on major branches. B) Overview of recombination events detected amongst alphavirus members using different detection methods (RDP, MaxChi, Chimaera, SciScan and 3Seq). C) Estimated p-values of detected recombination events using different detection methods. Nucleotide position corresponds to that of ZRU099/17. SFV Semliki Forest Virus (Genbank accession number CAA27742); nt = nucleotide. UPGMA: unweighted pair group method with arithmetic mean.
(DOCX)
