# Supplementary material for: Detection and genome characterization of Middelburg virus strains isolated from CSF and whole blood samples of humans with neurological manifestations in South Africa
Source: PLoS Negl Trop Dis. 2022 Jan 3;16(1):e0010020. doi: 10.1371/journal.pntd.0010020 (PMC8722727; doi:10.1371/journal.pntd.0010020)
Supplement: S1 Text — (DOCX) [file pntd.0010020.s001.docx]

**S1 Text:** Methods

Viral detection and Sanger sequencing

RNA was extracted from CSF and EDTA whole blood using the QIAmp viral RNA Kit, (Qiagen, Valencia, CA, USA) as per manufacturer’s instructions. Extracted RNA was subjected to screening using a published nested real-time alphavirus PCR with probes specific for Middelburg and Sindbis virus [1,2]. Primers/probes used in the current study are summarized in S1 Table. Products were also visualised on an agarose gel to detect all alphaviruses by the generic alphavirus primers. For differential diagnosis all MIDV positive specimens were also screened for flaviviruses and Shuni virus [3,4] and the veterinary students were also tested on a macro array chip for common and zoonotic causes of febrile and neurological disease [5]. In order to improve phylogenetic analysis a larger fragment of 347 base pairs (bp) of the nsP4 gene was amplified using MIDV specific primers [6] for all MIDV real time PCR positive samples.

A 550 bp fragment of the MIDV E1 gene was also amplified using a combination of newly designed and previously published[1] primers to investigate recombination events. First round PCR consisted of 10 µl RNA, Superscript Reaction Mix (Thermo Fisher Scientific, Massachusetts, USA), 0.4 µM forward and reverse primers (MIDV EF and MIDV E 1R), SuperScript III RT/Platinum Taq Mix and RNase free ddH_2_0 in a final volume of 50uL. Cycling conditions: 50°C for 30 min, 94° for 2min, (94°C for 15s, 52°C for 30 s, 68°C for 1min) x40; 68°C for 5 min, 4°C hold.

The nested MIDV E PCR reaction consisted of 2 µl DNA product from the first round amplification, DreamTaq Buffer (Thermo Fisher Scientific), 0.4 mM dNTP mix, 0.2 µM forward and reverse primers (MIDV EN 9F and MID10911 EN- 5’), DreamTaq Polymerase and RNase free ddH_2_0 in a final volume of 50uL. Cycling conditions: 95°C for 2min, (95°C for 30 s, 52°C for 30 s,72°C for 1min) x40 cycles, 72°C for 7 min, 4°C hold.

PCR products were purified using the Zymoclean™ Gel DNA recovery kit (Zymo Research, Irvine, USA) according to the manufacturer’s instructions and sent to Inqaba Biotechnology, South Africa for Sanger sequence analysis using the ABI PRISM 3100/3130 Genetic Analyser.

Virus isolation and Sequence-Independent Single-Primer Amplification (SISPA) with Rapid Amplification of cDNA Ends (RACE)

Virus isolation from PCR positive CSF or EDTA blood samples was attempted using Vero E6 cells in the BSL-3 facility at the University of Pretoria’s Centre for Viral Zoonosis (CVZ). Cells were cultured in Eagle’s Minimum Essential Medium (EMEM, Sigma Aldrich, Missouri, USA), supplemented with 10 % (v/v) Fetal Bovine Serum (FBS, Sigma Aldrich) and 2 % (v/v) MycoZap (Lonza, Basel, Switzerland) at 37°C and 5 % CO_2_ in 25 cm^2^ flasks. Near-confluent (80 %) monolayers were washed and inoculated with 200 µl of CSF or EDTA blood for 1h at 37°C before addition of 5 ml EMEM with 2 % FBS. Cells were monitored for CPE (cytopathic effect) over a 7 to 10-day period. Subsequent passaging was performed, and supernatant used to inoculate a 75 cm^2^ tissue culture flask in triplicate for preparation of stock virus and full genome sequencing. Virus culture supernatant was filtered through a Minisart 0.45 µm filter (Sartorius Stedim Biotech, Göttingen, Germany) and virus particles concentrated using an ultra-15 centrifugal filter with 10kDa molecular weight cut-off (Amicon, Merck, Darmstadt, Germany). Trizol-LS (Life Technologies, Waltham, MA, USA) was added to concentrated supernatant at a ratio of 500 µl supernatant to 1500 µl Trizol-LS. RNA was extracted using a column-based kit (Direct-Zol RNA kit, Zymo Research) including the in-column DNase treatment followed by purification using the RNA Clean and Concentrator-5 kit (Zymo Research) as per manufacturer’s instructions. RNA was converted to cDNA and amplified using the modified SISPA (RACE) technique described previously to enhance coverage of terminal ends [7].

References

1. van Niekerk S, Human S, Williams J, van Wilpe E, Pretorius M, Swanepoel R, et al. Sindbis and Middelburg Old World Alphaviruses Associated with Neurologic Disease in Horses, South Africa. Emerg Infect Dis. 2015;21(12):2225-9.

2. Sánchez-Seco MP, Rosario D, Quiroz E, Guzmán G, Tenorio A. A generic nested-RT-PCR followed by sequencing for detection and identification of members of the alphavirus genus. Journal of virological methods. 2001;95:153–61.

3. Zaayman D, Human S, Venter M. A highly sensitive method for the detection and genotyping of West Nile virus by real-time PCR. J Virol Methods. 2009;157(2):155-60.

4. Van Eeden C, Zaayman D, Venter M. A sensitive nested real-time RT-PCR for the detection of Shuni virus. J Virol Methods. 2014;195:100-5.

5. Venter M, Zaayman D, Niekerk S, Stivaktas V, Goolab S, Weyer J, et al. Macroarray assay for differential diagnosis of meningoencephalitis in southern Africa. J Clin Virol. 2014;60.

6. Steyn J. Culicoides midges (Diptera: Ceratopogonidae) as potential vectors for neurological arboviruses and the prevalence of infection at the wildlife/livestock/human interface. Pretoria, South Africa: University of Pretoria; 2019.

7. Jansen van Vuren P, Wiley M, Palacios G, Storm N, McCulloch S, Markotter W, et al. Isolation of a Novel Fusogenic Orthoreovirus from Eucampsipoda africana Bat Flies in South Africa. Viruses. 2016;8(3):65.
