# Supplementary material for: Detection and genome characterization of Middelburg virus strains isolated from CSF and whole blood samples of humans with neurological manifestations in South Africa
Source: PLoS Negl Trop Dis. 2022 Jan 3;16(1):e0010020. doi: 10.1371/journal.pntd.0010020 (PMC8722727; doi:10.1371/journal.pntd.0010020)
Supplement: S1 Table — (DOCX) [file pntd.0010020.s002.docx]

**S1 Table**: Primers and probes used for detection of alphaviruses targeting a conserved region of the nsP4 gene in alphaviruses and the E1 region of Middelburg virus specifically. Nucleotide positions are indicated for MIDV (Genbank accession number KF680222.1) and Sindbis virus (Genbank accession number U38305) genomes.

| PCR | Primer name (orientation/channel dye) | Sequence 5’-3’ | Position (MIDV/SINV) | Region and Amplicon size | Reference |
| --- | --- | --- | --- | --- | --- |
| Alpha first round | Alpha1+ (sense) | GAYGCITAYYTIGAYATGGTIGAIGG | 5888-6368/  6162-6642 | nsP4 480 bp | Sánchez-Seco et al. 2001[1] |
|  | Alpha1- (antisense) | KYTCYTCIGTRTGYTTIGTICCIGG |  |  |  |
| Alpha nested | Alpha2+ (sense) | GIAAYTGYAAYGTIACICARATG | 6066-6264/  6340-6538 | nsP4 198 bp |  |
|  | Alpha2- (antisense) | GCRAAIARIGCIGCIGCYYTIGGICC |  |  |  |
|  | MIDV probe | GCTTTAAGAAGTACGCATGCAACA | 6132-6155 | nsP4 N/A | van Niekerk et al. 2015[2] |
|  | SINV probe | ATGACGAGTATTGGGAGGAGTTTG | 6427-6450 |  |  |
| Nested specific MIDV | MNF (sense | GCAGCCTTTTGTCCGTCYAA | 5936-6283 | nsP4 347bp | Steyn et al. 2020[3] |
|  | MNR (antisense) | GGCTTCAAGTCRTAGGTTT |  |  |  |
| Nested specific SINV | SNF (sense) | GCAACCTTYTGCCCCGCYAA | 6209-6556 | nsP4 347 bp |  |
|  | SNR (antisense) | GGGACCAAATTATRCGTCT |  |  |  |
| MIDV E first round | MIDV EF (sense) | TTGTCAACGGAGAGAGCAC | 10231-11048 | E1 817 bp | This study |
|  | MIDV ER (antisense) | CTATGGGCGGAGCTACTGTG |  |  |  |
| MIDV E nested | MIDV EN 9F (sense) | ACCGGGTAGATTTGGGGACT | 10379-10930 | E1 551 bp | This study |
|  | MIDV 10911 EN (antisense) | CACTTTGCTGTGCAAGTGGT |  |  | van Niekerk et al.2015[2] |

References

1. Sánchez-Seco MP, Rosario D, Quiroz E, Guzmán G, Tenorio A. A generic nested-RT-PCR followed by sequencing for detection and identification of members of the alphavirus genus. Journal of virological methods. 2001;95:153–61.

2. van Niekerk S, Human S, Williams J, van Wilpe E, Pretorius M, Swanepoel R, et al. Sindbis and Middelburg Old World Alphaviruses Associated with Neurologic Disease in Horses, South Africa. Emerg Infect Dis. 2015;21(12):2225-9.

3. Steyn J, Fourie I, Steyl J, Williams J, Stivaktas V, Botha E, et al. Zoonotic Alphaviruses in Fatal and Neurologic Infections in Wildlife and Nonequine Domestic Animals, South Africa. Emerg Infect Dis. 2020;26(6):1182-91.
