# Supplementary material for: Detection and genome characterization of Middelburg virus strains isolated from CSF and whole blood samples of humans with neurological manifestations in South Africa
Source: PLoS Negl Trop Dis. 2022 Jan 3;16(1):e0010020. doi: 10.1371/journal.pntd.0010020 (PMC8722727; doi:10.1371/journal.pntd.0010020)
Supplement: S2 Table — Sequences used in the current study are in bold font. (DOCX) [file pntd.0010020.s003.docx]

**S2 Table:** GenBank and NCBI Sequence read archive accession numbers for sequence data for members of the *Alphavirus* genus used for phylogenetic analysis in this study. Sequences used in the current study are in bold font.

| Antigenic complex | Species | Host | Location | GenBank and NCBI sequence read archive accession numbers (strain name and gene where applicable) |
| --- | --- | --- | --- | --- |
| Reference strains | | | | |
| Middelburg | Middelburg virus | Horse | South Africa | KF680222.1 (SAE25/11) |
|  |  | Horse | Zimbabwe | EF536323.1 (857) |
|  |  | *Ae. vittatus* | CAR | KM115530.1 (ArB-8442) |
|  |  | *Am. variegatum* | CAR | KM115531.1 (ArTB-5290) |
| Western equine encephalitis | Sindbis virus | Human | Finland | JQ771797.1 |
|  |  | Arthropod | CAR | MF409177.1 |
|  | Whataroa virus | *Cx. Spp* | New Zealand | HM147993.1 |
| Eastern equine encephalitis | EEEV Lin I NA |  | USA | EF151502.1 |
|  | EEEV Lin II SA | Mosquito | Peru | DQ241303.1 |
|  | EEEV Lin III SA | Mosquito | Peru | DQ241304.1 |
| Venezuelan Equine Encephalitis | Venezuelan Equine Encephalitis virus | Mosquito | Venezuela | L04653.1 |
|  |  | Mosquito | Venezuela | L04653 |
|  | Everglades | *Cx melanoconion sp* | USA | AF075251.1 |
|  | Mucambo virus |  | Brazil | AF075253.1 |
|  | Tonate virus |  | Guyana | AF075254.1 |
| Barmah Forest | Barmah Forest virus | *Cx. annulirostris* | Australia | MK697274.1 |
| Ndumu | Ndumu virus | *Ae. Spp* | Africa | NC_016959.1 |
| Semliki Forest | Semliki Forest virus | *Ae. abnormalis group* | Africa | X04129.1 |
|  | Chikungunya virus | Human | Africa | AF369024.2 |
|  | O'nyong-nyong virus | Human | Uganda | AF079456.1 |
|  | Mayaro virus | Human | French Guiana | KJ013266.2 |
| Trocara | Trocara virus | *Cx. serratus* | Brazil | HM147991.1 |
| Eilat | Eilat virus | *An. coustani* | Israel | NC_018615.1 |
| Sleeping disease | Sleeping disease virus | Rainbow trout | France | AJ316246 |
| Southern elephant seal | Southern elephant seal virus | Seal | Australia | NC_016960.1 |
| **Current study** | | | | |
| Middelburg | Middelburg virus | **Human** | South Africa | **MN967314 and** **SAMN22891582 (ZRU099/17 full genome and raw sequence data)**  **MN967313 and** **SAMN22891583 (ZRUH399/17 full genome and raw sequence data**)  **MT264776 (ZRUH177/17 nsP4)**  **MT264777 (ZRUH248/17 nsP4)**  **MT264774 (ZRUH177/17 E1)**  **MT264775 (ZRUH248/17 E1)** |

*Cx. = Culex; Ae. = Aedes; An. = Anopheles*
