# Supplementary material for: Detection and genome characterization of Middelburg virus strains isolated from CSF and whole blood samples of humans with neurological manifestations in South Africa
Source: PLoS Negl Trop Dis. 2022 Jan 3;16(1):e0010020. doi: 10.1371/journal.pntd.0010020 (PMC8722727; doi:10.1371/journal.pntd.0010020)
Supplement: S3 Table — (DOCX) [file pntd.0010020.s004.docx]

**S3 Table**: Pathogens detected on the Chipron macroarray [1]

| 1. West Nile virus | 12. Varicella zoster virus | 23. *Leptospira spp.* |
| --- | --- | --- |
| 2. Rift valley fever | 13. Rabies virus | 24. *Mycobacterium tuberculosis* |
| 3. Chikungunya | 14. Epstein-barr virus | 25. *Ehrlichia spp*. |
| 4. Sindbis | 15. JC-virus | 26. *Neisseria meningitidis* |
| 5. Rubella | 16. Enterovirus | 27. *Plasmodium falciparum* |
| 6. Crimean Congo Haemorrhagic Fever | 17. Dengue virus | 28. Flavivirus genus I |
| 7. Cytomegalovirus | 18. *Rickettsia spp.* | 29.Flavivirus genus II |
| 8. Measles virus | 19. *Borerelia burgdorferi/garinii* | 30. Hepatitis A virus |
| 9. Mumps virus | 20. *Brucella spp.* | 31. Hepatitis B virus |
| 10. Herpes simplex virus 1 | 21. Adenovirus |  |
| 11. Herpes simplex virus 2 | 22. *Coxiella burnetti* |  |

References

1. Venter M, Zaayman D, Niekerk S, Stivaktas V, Goolab S, Weyer J, et al. Macroarray assay for differential diagnosis of meningoencephalitis in southern Africa. J Clin Virol. 2014;60.
