# Supplementary material for: Detection and genome characterization of Middelburg virus strains isolated from CSF and whole blood samples of humans with neurological manifestations in South Africa
Source: PLoS Negl Trop Dis. 2022 Jan 3;16(1):e0010020. doi: 10.1371/journal.pntd.0010020 (PMC8722727; doi:10.1371/journal.pntd.0010020)
Supplement: S4 Table — Nucleotide and amino acid identities are shown for complete (concatenated) genomes with amino acids in the lower left matrix and nucleotides in the upper right matrix. Only amino acid identities are shown for individual proteins. (DOCX) [file pntd.0010020.s005.docx]

**S4 Table**: Percentage identity of human Middelburg virus isolate full genomes and individual proteins to previously identified Middelburg virus strains. Nucleotide and amino acid identities are shown for complete (concatenated) genomes with amino acids in the lower left matrix and nucleotides in the upper right matrix. Only amino acid identities are shown for individual proteins.

| Strain | MIDV SAE25/11 | MIDV 857 | MIDV ArB-8422 | MIDV ArTB-5290 | ZRU099/17 | ZRUH399/17 |
| --- | --- | --- | --- | --- | --- | --- |
| Concatenated ORF 1 and 2 | | | | | | |
| MIDV SAE25/11 |  | 98,63% | 97,75% | 98,61% | 99,38% | 99,46% |
| MIDV 857 | 99,37% |  | 98,33% | 99,02% | 98,64% | 98,54% |
| MIDV ArB-8422 | 99,35% | 99,48% |  | 98,37% | 97,74% | 97,71% |
| MIDV ArTB-5290 | 99,37% | 99,51% | 99,54% |  | 98,53% | 98,52% |
| ZRU099/17 | 99,48% | 99,51% | 99,43% | 99,45% |  | 99,31% |
| ZRUH399/17 | 99,51% | 99,48% | 99,45% | 99,48% | 99,84% |  |
| nsp1 | | | | | | |
| MIDV SAE25/11 |  |  |  |  |  |  |
| MIDV 857 | 99,26% |  |  |  |  |  |
| MIDV ArB-8422 | 98,88% | 98,88% |  |  |  |  |
| MIDV ArTB-529 | 99,07% | 99,07% | 98,87% |  |  |  |
| ZRU099/17 | 99,07% | 99,44% | 100,00% | 98,88% |  |  |
| ZRUH399/17 | 99,26% | 98,88% | 100,00% | 98,70% | 98,70% |  |
| nsp2 | | | | | | |
| MIDV SAE25/11 |  |  |  |  |  |  |
| MIDV 857 | 99,37% |  |  |  |  |  |
| MIDV ArB-8422 | 99,62% | 99,25% |  |  |  |  |
| MIDV ArTB-529 | 99,75% | 99,37% | 99,62% |  |  |  |
| ZRU099/17 | 99,50% | 98,87% | 99,12% | 99,25% |  |  |
| ZRUH399/17 | 99,25% | 98,62% | 98,87% | 99,00% | 98,75% |  |
| nsp3 | | | | | | |
| MIDV SAE25/11 |  |  |  |  |  |  |
| MIDV 857 | 99,14% |  |  |  |  |  |
| MIDV ArB-8422 | 98,92% | 99,35% |  |  |  |  |
| MIDV ArTB-529 | 99,35% | 99,78% | 99,57% |  |  |  |
| ZRU099/17 | 99,57% | 98,71% | 98,49% | 99,57% |  |  |
| ZRUH399/17 | 99,35% | 98,49% | 98,28% | 98,49% | 98,92% |  |
| nsp4 | | | | | | |
| MIDV SAE25/11 |  |  |  |  |  |  |
| MIDV 857 | 99,50% |  |  |  |  |  |
| MIDV ArB-8422 | 99,70% | 99,80% |  |  |  |  |
| MIDV ArTB-529 | 99,70% | 99,80% | 100,00% |  |  |  |
| ZRU099/17 | 99,50% | 99,30% | 99,50% | 99,50% |  |  |
| ZRUH399/17 | 99,80% | 99,70% | 99,80% | 99,80% | 99,70% |  |
|  | | | | | | |
| capsid | | | | | | |
| MIDV SAE25/11 |  |  |  |  |  |  |
| MIDV 857 | 98,90% |  |  |  |  |  |
| MIDV ArB-8422 | 99,30% | 99,60% |  |  |  |  |
| MIDV ArTB-529 | 99,30% | 99,60% | 100,00% |  |  |  |
| ZRU099/17 | 99,30% | 98,20% | 98,50% | 98,50% |  |  |
| ZRUH399/17 | 99,30% | 98,20% | 98,50% | 98,50% | 99,30% |  |
| E3 | | | | | | |
| MIDV SAE25/11 |  |  |  |  |  |  |
| MIDV 857 | 100,00% |  |  |  |  |  |
| MIDV ArB-8422 | 100,00% | 100,00% |  |  |  |  |
| MIDV ArTB-529 | 100,00% | 100,00% | 100,00% |  |  |  |
| ZRU099/17 | 100,00% | 100,00% | 100,00% | 100,00% |  |  |
| ZRUH399/17 | 98,50% | 98,50% | 98,50% | 98,50% | 98,50% |  |
| E2 | | | | | | |
| MIDV SAE25/11 |  |  |  |  |  |  |
| MIDV 857 | 99,30% |  |  |  |  |  |
| MIDV ArB-8422 | 99,00% | 99,80% |  |  |  |  |
| MIDV ArTB-529 | 99,00% | 99,80% | 100,00% |  |  |  |
| ZRU099/17 | 99,00% | 98,80% | 98,60% | 98,60% |  |  |
| ZRUH399/17 | 99,80% | 99,50% | 99,30% | 99,30% | 99,30% |  |
| 6K | | | | | | |
| MIDV SAE25/11 |  |  |  |  |  |  |
| MIDV 857 | 100,00% |  |  |  |  |  |
| MIDV ArB-8422 | 100,00% | 100,00% |  |  |  |  |
| MIDV ArTB-529 | 98,40% | 98,40% | 98,40% |  |  |  |
| ZRU099/17 | 98,40% | 98,40% | 98,40% | 96,70% |  |  |
| ZRUH399/17 | 100,00% | 100,00% | 100,00% | 98,40% | 98,40% |  |
| E1 | | | | | | |
| MIDV SAE25/11 |  |  |  |  |  |  |
| MIDV 857 | 99,77% |  |  |  |  |  |
| MIDV ArB-8422 | 99,54% | 99,77% |  |  |  |  |
| MIDV ArTB-529 | 99,09% | 99,32% | 99,09% |  |  |  |
| ZRU099/17 | 99,54% | 99,77% | 99,54% | 99,09% |  |  |
| ZRUH399/17 | 99,54% | 99,77% | 99,54% | 99,09% | 99,54% |  |

MIDV: Middelburg virus
