# Supplementary material for: Detection and genome characterization of Middelburg virus strains isolated from CSF and whole blood samples of humans with neurological manifestations in South Africa
Source: PLoS Negl Trop Dis. 2022 Jan 3;16(1):e0010020. doi: 10.1371/journal.pntd.0010020 (PMC8722727; doi:10.1371/journal.pntd.0010020)
Supplement: S5 Table — Numbering refers to sequence positions of isolate SAE25/11. Changes in ZRU099/17 and ZRUH399/17 as compared to MIDV SAE25/11 are highlighted in grey. (DOCX) [file pntd.0010020.s006.docx]

**S5 Table**: Amino acid comparisons of the structural and non-structural proteins between different Middelburg virus strains. Only positions where changes occurred as compared to MIDV SAE25/11 are indicated. Numbering refers to sequence positions of isolate SAE25/11. Changes in ZRU099/17 and ZRUH399/17 as compared to MIDV SAE25/11 are highlighted in grey.

| Region | **nsP1** | | | | | | | | | | | | | | | | **nsP2** | |
| --- | --- | --- | --- | --- | --- | --- | --- | --- | --- | --- | --- | --- | --- | --- | --- | --- | --- | --- |
| Amino acid position | 31 | 36 | 79 | 146 | 189 | 235 | 286 | 325 | 430 | 461 | 485 | 497 | 512 | 515 | 520 | 532 | 19 | 46 |
| MIDV SAE25/11 | A | P | A | S | V | L | G | G | T | G | P | D | P | S | T | D | T | E |
| MIDV 857 | A | P | A | N | A | L | G | G | I | G | P | D | P | T | T | D | T | D |
| MIDV ArB-8422 | A | S | A | N | V | F | G | G | T | G | P | N | P | T | A | D | T | E |
| MIDV ArTB-5290 | V | P | T | N | V | L | G | G | T | W | P | D | P | T | T | D | M | E |
| ZRU099/17 | A | P | A | N | A | L | R | G | T | G | P | D | P | T | T | E | T | E |
| ZRUH399/17 | A | P | A | N | V | L | G | A | T | G | H | D | H | S | T | D | T | E |
| Region | **nsP2** | | | | | | | | | | | | | | | | **nsP3** | |
| Amino acid position | 95 | 99 | 269 | 299 | 323 | 430 | 470 | 484 | 514 | 585 | 665 | 673 | 678 | 719 | 756 | 791 | 49 | 249 |
| MIDV SAE25/11 | E | V | V | D | T | P | P | E | P | I | I | P | R | G | V | G | T | T |
| MIDV 857 | E | M | V | N | T | P | P | K | P | I | I | S | R | G | V | G | T | T |
| MIDV ArB-8422 | E | M | V | D | T | P | P | E | P | V | V | P | R | G | V | G | N | T |
| MIDV ArTB-5290 | E | M | V | D | T | P | P | E | P | I | I | P | R | G | V | G | T | T |
| ZRU099/17 | Q | V | M | D | K | P | P | E | P | I | I | P | R | G | V | R | T | T |
| ZRUH399/17 | E | V | V | D | T | S | H | E | H | I | I | P | S | D | M | G | T | N |
| Region | **nsP3** | | | | | | | | | **nsP4** | | | | **Capsid** | | | | |
| Amino acid position | 271 | 360 | 365 | 367 | 378 | 387 | 439 | 445 | 449 | 30 | 242 | 374 | 515 | 4 | 65 | 75 | 85 | 102 |
| MIDV SAE25/11 | V | L | V | K | T | S | N | G | A | I | R | F | T | V | I | P | P | P |
| MIDV 857 | V | L | V | E | A | R | Y | G | A | V | Q | F | T | I | T | S | P | P |
| MIDV ArB-8422 | I | L | V | E | A | R | N | G | A | V | Q | F | T | I | T | P | P | P |
| MIDV ArTB-5290 | V | L | V | E | A | R | N | G | A | V | Q | F | T | I | T | P | P | P |
| ZRU099/17 | V | L | L | K | T | S | N | W | A | I | Q | L | R | V | I | P | L | P |
| ZRUH399/17 | V | M | V | K | T | S | N | G | S | I | Q | F | T | V | I | P | P | H |
| Region | **Capsid** | **E3** | **E2** | | | | | | | **6K** | | **E1** | | | | | | |
| Amino acid position | 240 | 7 | 1 | 63 | 76 | 256 | 274 | 291 | 370 | 8 | 29 | 103 | 113 | 120 | 122 | 187 | 330 | 377 |
| MIDV SAE25/11 | G | A | W | A | E | P | A | T | A | A | F | L | V | A | Y | Q | I | T |
| MIDV 857 | G | A | G | A | E | P | T | T | V | A | F | L | V | A | Y | Q | V | T |
| MIDV ArB-8422 | G | A | G | S | E | P | T | T | V | A | F | L | V | A | Y | H | V | T |
| MIDV ArTB-5290 | G | A | G | S | E | P | T | T | V | A | L | Q | V | V | F | Q | V | T |
| ZRU099/17 | R | A | G | A | Q | L | T | R | A | P | F | L | V | A | Y | Q | V | K |
| ZRUH399/17 | R | D | G | A | E | P | T | T | A | A | F | L | M | A | Y | Q | V | T |
