# Supplementary material for: Detection and genome characterization of Middelburg virus strains isolated from CSF and whole blood samples of humans with neurological manifestations in South Africa
Source: PLoS Negl Trop Dis. 2022 Jan 3;16(1):e0010020. doi: 10.1371/journal.pntd.0010020 (PMC8722727; doi:10.1371/journal.pntd.0010020)
Supplement: S6 Table — Only positions where changes occurred as compared to MIDV SAE25/11 are indicated. Numbering refers to sequence positions of isolate SAE25/11. Changes in ZRU099/17 and ZRUH399/17 as compared to MIDV SAE25/11 are highlighted in grey. Gaps and inserts are indicated with “-” and “^” symbols, respectively. (DOCX) [file pntd.0010020.s007.docx]

**S6 Table**: Nucleotide acid comparisons of the 5’ and 3’ non-coding regions between different Middelburg virus strains. Only positions where changes occurred as compared to MIDV SAE25/11 are indicated. Numbering refers to sequence positions of isolate SAE25/11. Changes in ZRU099/17 and ZRUH399/17 as compared to MIDV SAE25/11 are highlighted in grey. Gaps and inserts are indicated with “-” and “^” symbols, respectively.

| Region | **5'UTR** | **3'UTR** | | | | | | | | | | | | | | | | | | | | | | | | Length of genome bp |
| --- | --- | --- | --- | --- | --- | --- | --- | --- | --- | --- | --- | --- | --- | --- | --- | --- | --- | --- | --- | --- | --- | --- | --- | --- | --- | --- |
| Nucleotide position | 37 | 7 | 8 | 12 | 27 | 30 | 35 | 36 | 50 | 65 | 78 | 79 | 94 | 110 | 142^ | 143 | 144 | 145 | 198 | 214^ | 400 | 409 | 435 | 456 | 479 |  |
| MIDV SAE25/11 | A | T | T | C | T | G | A | C | G | T | G | T | T | T | - | A | C | C | C | - | T | A | T | A | T | 11674 |
| MIDV 857 | A | T | A | C | T | - | A | T | A | T | G | T | C | T | - | C | C | C | C | G | C | A | T | A | T | 11674 |
| MIDV ArB-8422 | C | T | A | C | C | - | T | T | A | C | A | C | C | C | A | C | C | C | T | G | C | T | T | G | C | 11550 |
| MIDV ArTB-5290 | A | C | A | T | T | - | A | T | A | T | G | T | C | T | - | C | T | T | C | G | C | A | C | A | T | 11468 |
| ZRU099/17 | A | T | A | C | T | - | A | C | G | T | G | T | T | T | - | C | C | C | C | - | T | A | T | A | T | 11673 |
| ZRUH399/17 | A | T | T | C | T | - | A | C | A | T | G | T | T | T | - | C | C | C | C | - | T | A | T | A | T | 11673 |
