# Supplementary material for: Detection and genome characterization of Middelburg virus strains isolated from CSF and whole blood samples of humans with neurological manifestations in South Africa
Source: PLoS Negl Trop Dis. 2022 Jan 3;16(1):e0010020. doi: 10.1371/journal.pntd.0010020 (PMC8722727; doi:10.1371/journal.pntd.0010020)
Supplement: S1 Fig — “_” indicates absent sequence. MIDV = Middelburg virus. (DOCX) [file pntd.0010020.s008.docx]

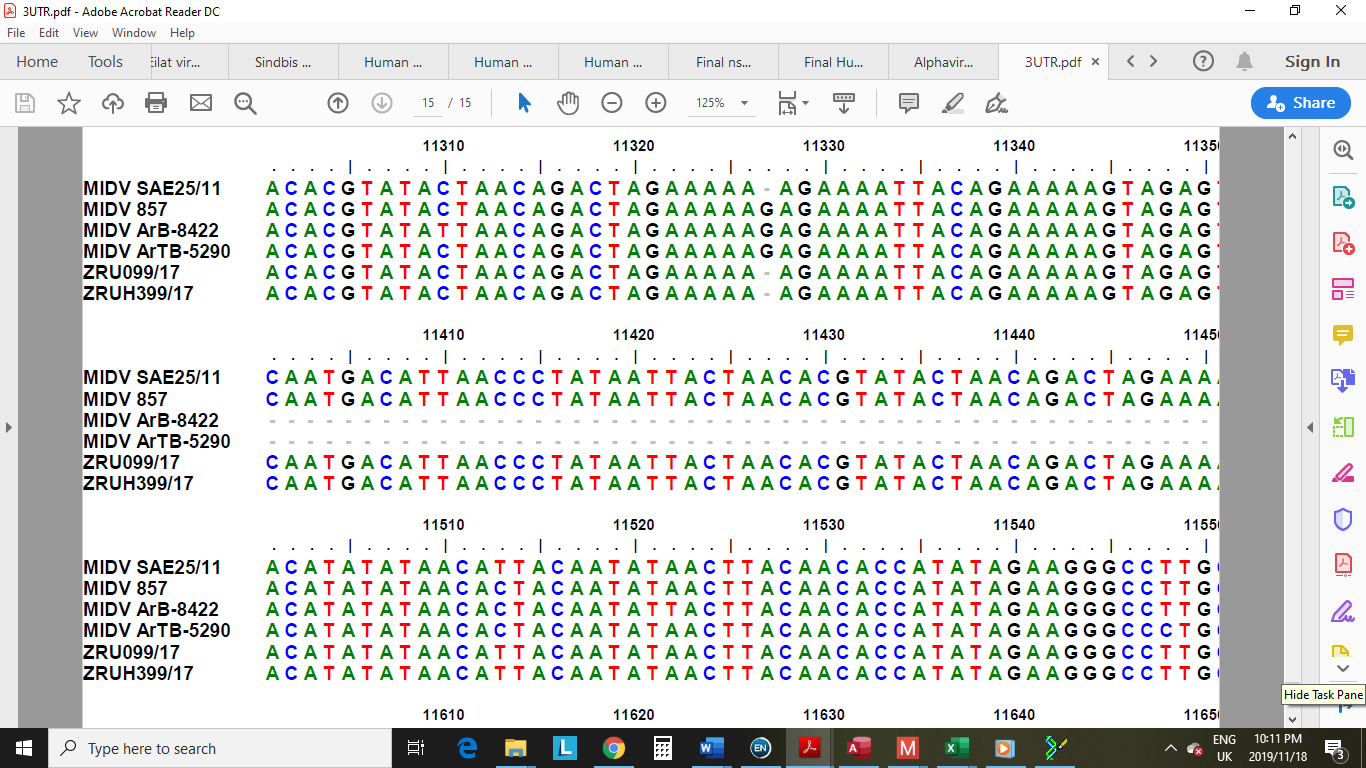


**S1 Figure**: The human MIDV strains, ZRU099/17 and ZRUH399/17 contain repeat sequences in the 3’UTR as observed in MIDV strains from horses (MIDV 857 and MIDV SAE25/11) but missing from historical arthropod strains (MIDV ArB-8422 and MIDV ArTB-5290) as indicated by the outlined box. “_” indicates absent sequence. MIDV= Middelburg virus.
