# Supplementary material for: Detection and genome characterization of Middelburg virus strains isolated from CSF and whole blood samples of humans with neurological manifestations in South Africa
Source: PLoS Negl Trop Dis. 2022 Jan 3;16(1):e0010020. doi: 10.1371/journal.pntd.0010020 (PMC8722727; doi:10.1371/journal.pntd.0010020)
Supplement: S2 Fig — A) UPMGA phylogenetic tree indicating the breakpoint region with probabilities shown on major branches. B) Overview of recombination events detected amongst alphavirus members using different detection methods (RDP, MaxChi, Chimaera, SciScan and 3Seq). C) Estimated p-values of detected recombination events using different detection methods. Nucleotide position corresponds to that of ZRU099/17. SFV Semliki Forest Virus (Genbank accession number CAA27742); nt = nucleotide. UPGMA: unweighted pair group method with arithmetic mean. (DOCX) [file pntd.0010020.s009.docx]

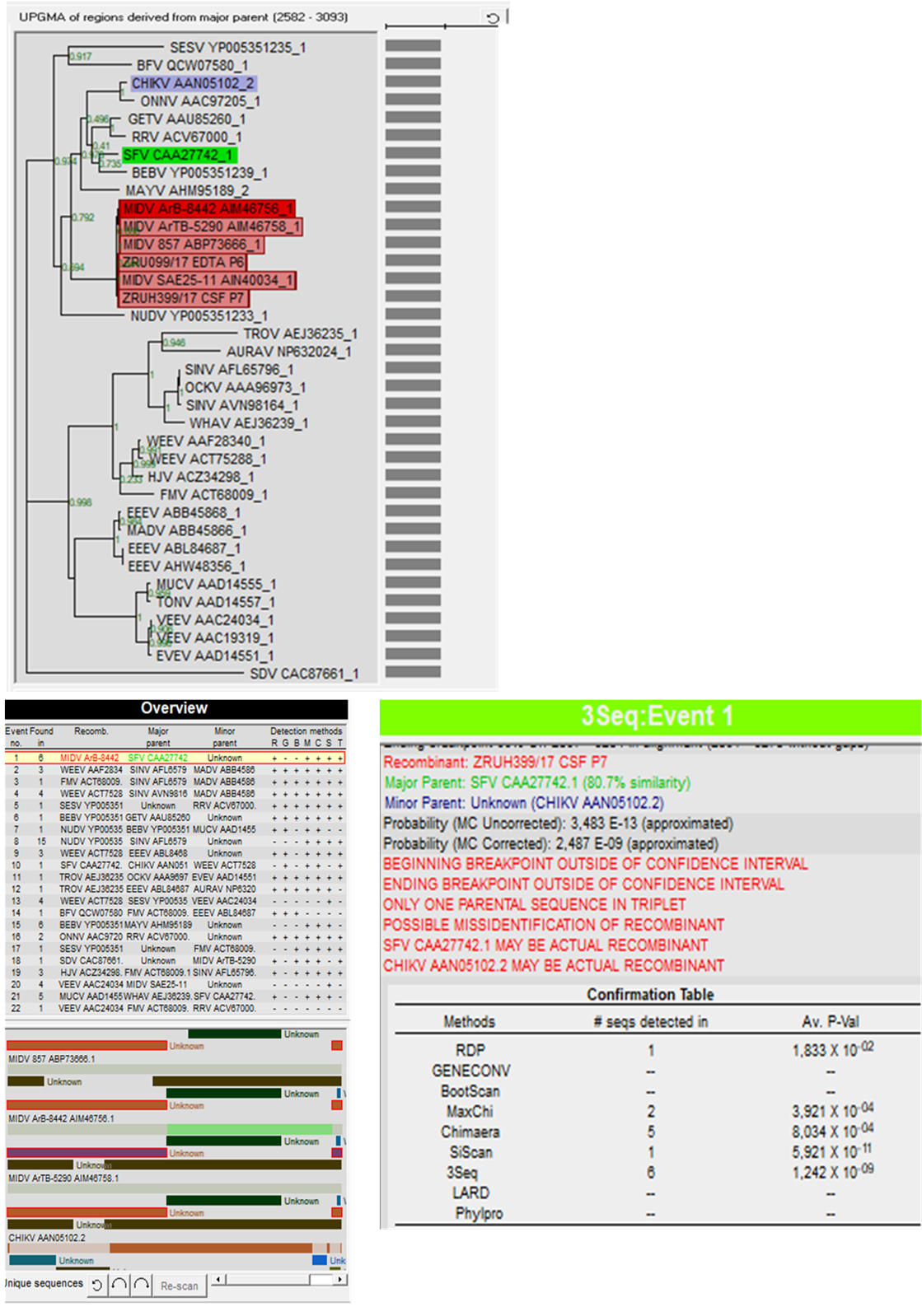


A)

B)

C)

**S2 Figure:** Screenshots of the identified possible recombination events associated with currently described MIDV full genomes (ZRU099/17 and ZRUH399/17)) within the structural region. A) UPMGA phylogenetic tree indicating the breakpoint region with probabilities shown on major branches. B) Overview of recombination events detected amongst alphavirus members using different detection methods (RDP, MaxChi, Chimaera, SciScan and 3Seq). C) Estimated p-values of detected recombination events using different detection methods. Nucleotide position corresponds to that of ZRU099/17. SFV Semliki Forest Virus (Genbank accession number CAA27742); nt= nucleotide. UPGMA: unweighted pair group method with arithmetic mean
